# Management of pericardial tamponade in the electrophysiology laboratory: results from a national survey

**DOI:** 10.1007/s00392-022-02042-x

**Published:** 2022-06-17

**Authors:** Andreas Metzner, Stephan D. Reubold, Sophie Schönhofer, Bruno Reißmann, Feifan Ouyang, Laura Rottner, Ruben Schleberger, Leon Dinshaw, Julia Moser, Fabian Moser, Marc Lemoine, Paula Münkler, Shinwan Kany, Daniel Steven, Philipp Sommer, Paulus Kirchhof, Andreas Rillig

**Affiliations:** 1 Department of Cardiology, University Heart and Vascular Center Hamburg, Martinistraße 52, 20246 Hamburg, Germany; 2 DZHK, Hamburg, Germany; 3 DZHK, Kiel, Germany; 4 DZHK, Lübeck, Germany; 5 University Heart Center, University of Cologne, Cologne, Germany; 6 Herz- Und Diabeteszentrum NRW, Bad Oeynhausen, Ruhr-University of Bochum, Bad Oeynhausen, Germany; 7 Institute of Cardiovascular Sciences, University of Birmingham, Birmingham, UK; 8 Asklepios Klinik Harburg, Hamburg, Germany

**Keywords:** Catheter ablation, Cardiac tamponade, Complication, Pericardial puncture, Survey, Electrophysiology, EP lab

## Abstract

**Background:**

Despite continued efforts to improve the safety of catheter ablation, pericardial tamponade remains one of its more frequent, potentially life-threatening complications. Management of cardiac tamponade is not standardized and uncertainties regarding acute treatment remain.

**Methods:**

This survey sought to evaluate the management of cardiac tamponade in German EP centers via a standardized postal questionnaire. All 341 identified German EP centers were invited to return a questionnaire on their standards for the management of cardiac tamponade.

**Results:**

A total of 189 German EP centers completed the questionnaire. Several precautions are followed to avoid pericardial tamponade: A minority of centers preclude very old patients (19%) or those with a high body mass index (30%) from ablation. Non-vitamin K antagonist oral anticoagulants are briefly paused in most centers (88%) before procedures, while vitamin K antagonists are continued. Pericardial tamponade is usually treated using reversal of heparin by applying protamine (86%) and pericardiocentesis under both, fluoroscopic and echocardiographic guidance (62%). A pigtail catheter is mostly inserted (97%) and autotransfusion of aspirated blood is performed in 47% of centers. The decision for surgical repair depends on different clinical and infrastructural aspects. The timing of reinitiation of anticoagulation widely differs between the centers. Approximately 1/3 of centers prescribe nonsteroidal anti-inflammatory agents, colchicine or steroids after pericardiocentesis.

**Conclusion:**

The present survey shows that the management of cardiac tamponade is still inhomogeneous in German ablation centers. However, multiple findings of this survey can be generalized and might guide especially less experienced operators and centers in their treatment and decision strategies.

## Introduction

Catheter ablation is a well-established strategy for the treatment of cardiac arrhythmias [1, 2] that is increasingly offered to elderly and multimorbid patients [3]. Despite substantial improvements in catheter technologies including imaging to guide transseptal puncture, contact force measurement, balloon-based devices and alternative energy sources, the number of periprocedural complications has even increased in the past years [3, 4]. Potential reasons might be patient selection with an increasing number of more complex procedures performed in older and multimorbid patients and a higher number of low-volume centers [3]. One of the most frequent, potentially life-threatening acute complications is cardiac tamponade with an incidence ranging from as low as 0.2% during catheter ablation of supraventricular tachycardias (SVT) up to 9.4% during ablation of epicardial ventricular tachycardia (VT [5], [6], [7]). Even if prompt and proper pericardiocentesis is performed by experienced and well-trained interventional electrophysiologists, mortality remains substantial depending on procedure type and patient characteristics [5, 7].

Management of cardiac tamponade is not standardized and uncertainties remain such as epicardial puncture technique, reversal of heparin effects, the use of autotransfusion and its modalities, timing of involvement of cardiac surgery in severe cases and postprocedural management [2].

This survey sought to evaluate the management of cardiac tamponade in German ablation centers via a standardized questionnaire including queries on precautions, periprocedural and postprocedural management of cardiac tamponade (questionnaire in supplement).

## Methods

This physician-based survey was conducted by sending out questionnaires to all 341 identified hospitals in Germany performing electrophysiological procedures as assessed via a white list. The questionnaire including 46 questions is shown in the supplementary material. The postal questionnaires were sent to all identified centers and up to two reminders were sent out, in case a center did not answer within 3 months. The results were obtained anonymously. The survey results are displayed as categorial values (numbers and proportions).

## Results

### Baseline data

**Fig. 1:**
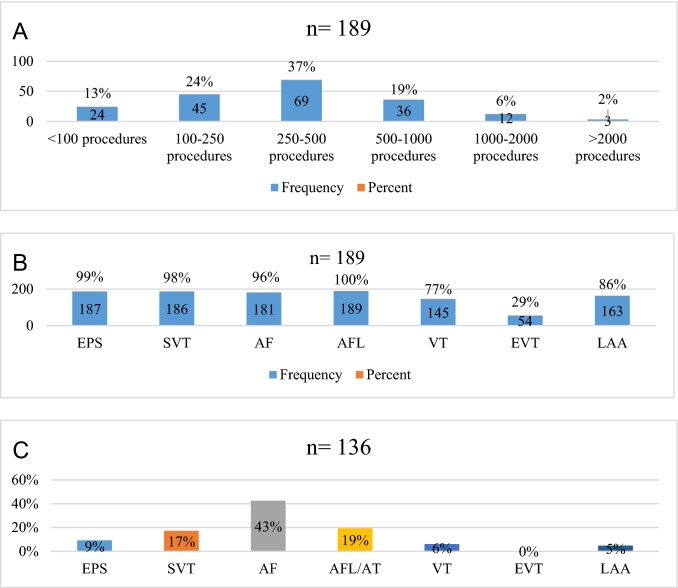
**A** Number of procedures in EP centers. **B** Type of procedures performed in EP centers. **C** Percentagewise distribution of procedures in EP centers

A total of 341 German ablation centers were identified and 189/341 (55%) datasets from responding centers were included into our analysis. An overview of the annual EP-procedure load of the centers is given in Fig. **1**, based on procedures performed in 2019. Noteworthy, 13% of participating centers perform less than 100 EP-procedures per year, another 2% more than 2000. While most centers reported to perform diagnostic EP examinations (187/189, 99%), SVT ablations (186/189, 98%), AF ablations (181/189, 96%) and AFL ablations (189/189, 100%), a total of 145/189 (77%) centers also report on endocardial VT ablations, 54/189 (29%) on epicardial VT ablations and 163/189 (86%) on left atrial appendage occluder implantation. With 43% of the total procedure load, AF ablations account for the majority of reported procedures, while epicardial VT ablation represents the least often performed intervention (< 1%; Fig. 1C).

With 133/189 (70%), many of the EP centers report on having dedicated EP laboratories. An overview of qualification levels of EP physicians is given in Suppl. Figure 1. Of note, 44% of centers have a dedicated EP nursing team.

The technical infrastructure in view of EP mapping and ablation platforms varies substantially between the centers. Radiofrequency current with or without a 3D mapping system is used in 174/189 (92%) and cryoballoon in 151/189 (80%) institutions. In contrast, laser balloon ablation is applied in only 9/189 (5%) of the centers, while 6/189 (3%) institutions report to use other ablation systems.

## Body mass index, age and international normalized ratio

In 132/189 (70%) centers, the body mass index (BMI) does not serve as an exclusion criterion for any kind of EP-procedure. However, 57/189 (30%) EP centers report BMI limits: 13/57 (23%) centers have fixed BMI limits for all kind of EP-procedures (BMI of ≥ 30.0 in 1/13 (8%) centers, ≥ 35.0 in 2/13 (15%) and > 40 in in 10/13 (77%) centers), while 44/57 (77%) centers have BMI limits only for left atrial and/or ventricular procedures. Of these, 1/44 (2%) centers state a BMI ≥ 34.9, 19/44 (43%) a BMI range of 35.0–39.9 and 21/44 (48%) centers a BMI ≥ 40 as an exclusion criterion. Another center has no fixed BMI limits, one center has only limits for AF procedures and one center did not further specify.

**Fig. 2:**
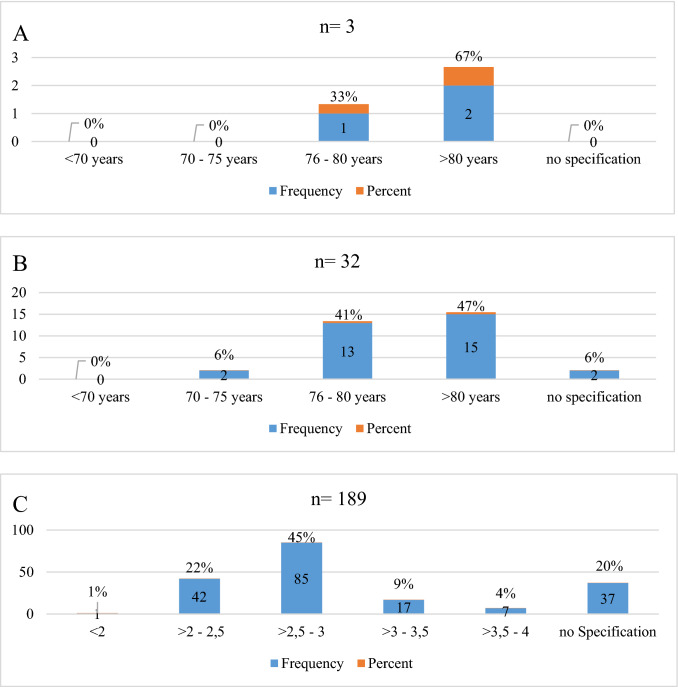
**A** Age limits for all EP-procedures. **B** Age limits for left atrial/ventricular EP-procedures. **C** INR limits for EP-procedures

Age as an exclusion criterion for EP-procedures was reported by 35/189(19%) centers. Age as well as INR limits of the centers are given in Fig. 2.

## Periinterventional anticoagulation management

### Preprocedural anticoagulation

Non-vitamin K antagonist oral anticoagulants (NOACs) are paused in 166/189 (88%) of the centers before the procedure, while 23/189 (12%) centers perform interventions on uninterrupted NOAC treatment. Among centers that withhold OAC, 28/166 (17%) pause OAC the day before, 41/166 (25%) pause the evening before and 85/166 (51%) pause on the day of the procedure. 8/166 (5%) centers apply other strategies and 4/166 (2%) do not further specify. Post-intervention OAC is started immediately following the procedure in 26/189 (14%) centers, after defined time ranges in 102/189 (54%) centers (Suppl. Figure 2) and the following day in 42/189 (22%). In 19/189 (10%) centers no information on postinterventional anticoagulation was provided.

### Transseptal puncture

An overview of imaging and monitoring modalities as well as the type of diagnostic catheters used for transseptal puncture by the EP centers is provided in Suppl. Figure 3. A total of 111/189 (59%) centers use fluoroscopy alone to guide transseptal puncture, while 56/189 (30%) use transesophageal echocardiography guidance, 12/189 (6%) intracardiac echocardiography and 6/189 (3%) use both imaging modalities; another 4/189 (2%) centers report on other imaging modalities for transseptal puncture (e.g., EP navigation/needle potential). Diagnostic catheters used for transseptal puncture are listed in Suppl. Figure 3B. While 115/189 (61%) of the centers perform transseptal puncture under pressure control, 74/189 (39%) do not.

Details on timing of heparin application and the periinterventional target activated clotting time (ACT) are provided in Suppl. Figure 4. Nineteen out of 189 (10%) centers reported weight-adapted application of heparin before transseptal puncture, and 87/189 (46%) centers thereafter. In 78/189 (41%) centers, only a certain amount of the total body weight-adapted heparin dose is administered prior to and the rest after transseptal puncture. 5/189 (2.6%) centers did not provide any information. The majority (71/78, 91%) of centers state that the amount of heparin before the transseptal puncture is ≤ 5000 units (I.U.), while 4/78 (5%) give > 5000 I.U. and 3/78 (4%) did not further specify. Regarding target ACT levels, 14/189 (7%) centers reported ≤ 250 s, 99/189 (52%) ACT levels > 250–300 s and 72/189 (38%) > 300 s during left atrial/ventricular procedures. No information was provided by 4/189 (2%) centers. Only 32/189 (17%) centers perform ACT measurement before, all others following transseptal puncture. Suppl. Figure 4 shows the intervals of the ACT measurements.

### Infrastructure and safety

The survey revealed that 61/189 (32%) of the EP centers have on site cardiac surgery, whereas 128/189 (67%) centers have not. All centers without cardiac surgery stated to collaborate with external cardiac surgery institutions.

Suppl. Figure 5A shows in how many centers and in which kind of procedures invasive blood pressure measurement is performed. Centers and types of procedures with non-invasive blood pressure measurements and the corresponding intervals are shown in Suppl. Figure 5B. The majority of EP centers (180/189, 95%) have echocardiography permanently available on site in the EP laboratory.

Echocardiography to rule out pericardial effusion following EP-procedures is routinely performed directly on the EP table in 151/189 (80%) centers as an institutional standard. Pericardial effusion is primarily or repeatedly ruled out in the recovery room and/or on the ward in 31/189 (16%) centers, 74/189 (39%) centers exclude a pericardial effusion the following day. A total of 26/189 (14%) centers perform an echocardiography again at the day of discharge and 3/189 (2%) centers only if clinically indicated (multiple answers possible). A total of 8/189 (4%) centers use different schemes. Suppl. Figure 6A illustrates postprocedural monitoring modalities and durations, if provided by the centers.

Of note, almost all centers (187/189, 98%) have a dedicated pericardiocentesis set prepared for emergencies in the cath lab and half of the centers (93/189, 49%) have regular trainings with the EP team to prepare for emergency intervention in case of cardiac tamponade.

### Management of acute pericardial tamponade

**Fig. 3:**
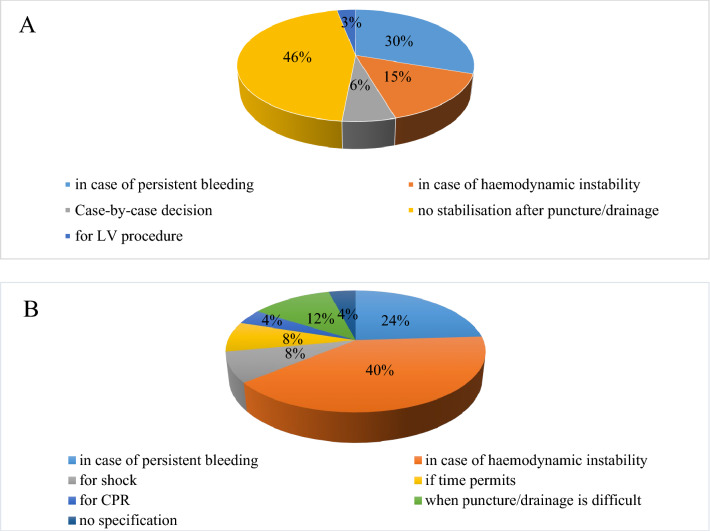
**A** Reasons for contacting cardiac surgery in case of cardiac tamponade. **B** Reasons for invasive blood pressure measurement in case of cardiac tamponade

Whenever pericardial tamponade occurs, 49/189 (26%) centers immediately contact an institutional resuscitation team. Another 25/189 (13%) centers always inform a cardiac surgeon in case of pericardial tamponade, whereas more than two-thirds of centers (131/189, 69%) do not. 33/189 (17%) centers inform the cardiac surgeon only in specific situations as listed in Suppl. Figure 6A. If not already present, 131/189 (69%) centers place an arterial line for invasive blood pressure measurement in case of pericardial tamponade, 33/189 (17%) do not. In 25/189 (13%) centers, an upgrade to invasive blood pressure management is only performed in certain instances (Fig. 3B).

### Pericardiocentesis

Fluoroscopy as the primary imaging modality for pericardiocentesis is used in 53/189 (28%) centers, whereas 16/189 (8%) centers use only echocardiography. 117/189 (62%) centers use both, echocardiography and fluoroscopy and another 3/189 (2%) centers do not use any imaging modality for pericardiocentesis. The fluoroscopic projections used by the centers are shown in Suppl. Figure 7.

While 145/189 (77%) centers insert an additional sheath into the pericardium after successful pericardial access is achieved, pigtail catheter insertion is reported by almost all centers (184/189, 97%) and only 5/189 (3%) do not use a pigtail catheter. The sizes of sheaths and pigtail catheters are provided in Suppl. Figure 8.

When cardiac tamponade occurs, protamine is administered on a routine basis in many centers [163/189 (86%)]. A third of the centers [60/163 (37%)] administers protamine immediately when cardiac tamponade is diagnosed, 31/163 (19%) centers after safe access to the pericardium is achieved and 53/163 (33%) once all blood is aspirated from the pericardial space. In 14/163 (9%) centers, individual factors such as persistent bleeding, high ACT levels, or the amount of effusion are applied criteria for administering protamine. Five out of 163 centers (3%) did not give any information. In case of protamine administration, 68/163 (42%) centers antagonize the previously administered heparin in a 1:1 ratio and 54/163 (33%) adapt the dosing according to the last ACT measured. 27/163 (17%) centers apply 5000 I.U. protamine as a standard dosage, 5/163 (3%) 3000 I.U. and 8/163 (5%) centers use other dosages. One center did not further specify.

**Fig. 4:**
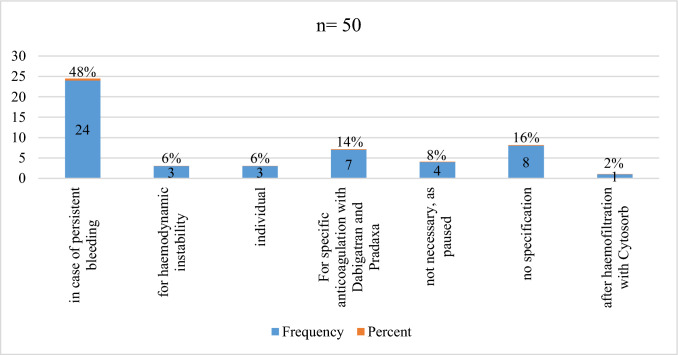
Specific situations in which an antidote is used in NOAC therapy in the event of pericardial tamponade

Overall, only 5% of the centers ever applied a specific NOAC antidote for adjunct treatment of cardiac tamponade. Accordingly, 179/189 (95%) centers have not used any antidote so far. 56/189 (30%) centers routinely administer clotting factors (PPSB, aPPSB, recombinant FVIIa). Almost two-thirds of the centers (122/189, 65%) do not give a NOAC antidote whereas 17/189 (9%) would do so. 50/189 (26%) centers would only use an antidote in certain instances as illustrated in Fig. 4.

With regard to autotransfusion of aspirated blood, 36/189 (19%) centers responded that they reinfuse blood only before protamine administration, while 34/189 (18%) centers also autotransfuse after protamine administration. However, more than half (101/189, 53%) of the centers do not perform autotransfusion. 15/189 (10%) centers report other approaches. Three centers (2%) did not answer. When autotransfusion is performed, 31/70 (44%) do not use a blood filter, 17/70 (24%) use a blood filter, 4/70 (6%) reinfuse blood via a Cellsafer and 18/70 (26%) did not further specify.

### Decision for cardiac surgery

**Fig. 5:**
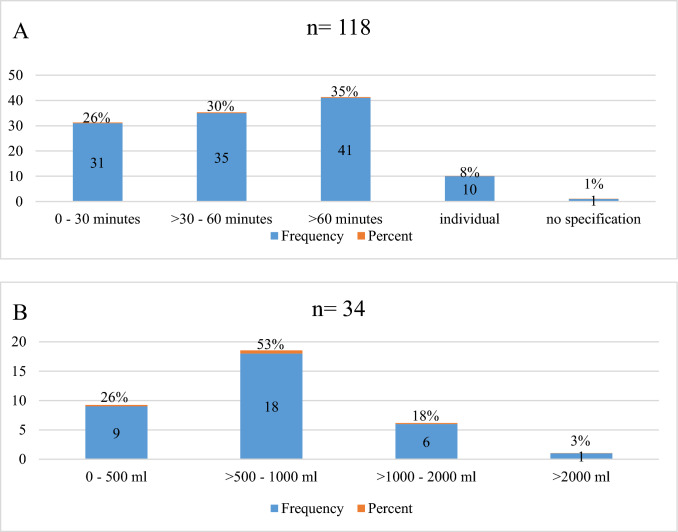
**A** Time range for the decision for cardiosurgical treatment after implementation of all conventional measures. **B** Minimum amount of aspirated blood and further trailing effusion for the decision for cardiosurgical care after implementation of all conventional measures

Most of the centers [118/189 (62%)] decide for cardiac surgical treatment if the bleeding does not stop after all conventional treatment options within certain periods of time were applied (Fig. 5A). 34/189 (18%) centers decide after a certain amount of blood was aspirated and bleeding continues (Fig. 5B) and 37/189 (20%) centers have a different approach, not further specified.

### Subsequent surveillance and monitoring

Once pericardial tamponade is successfully treated, 151/189 (80%) centers monitor their patients on an Intensive Care Unit (ICU), another 26/189 (14%) centers on an Intermediate Care Unit, and 9/189 (5%) might use both the ICU or the Intermediate Care Unit. Only 2/189 (1%) centers monitor patients after cardiac tamponade on regular wards and one single center did not further specify.

**Fig. 6:**
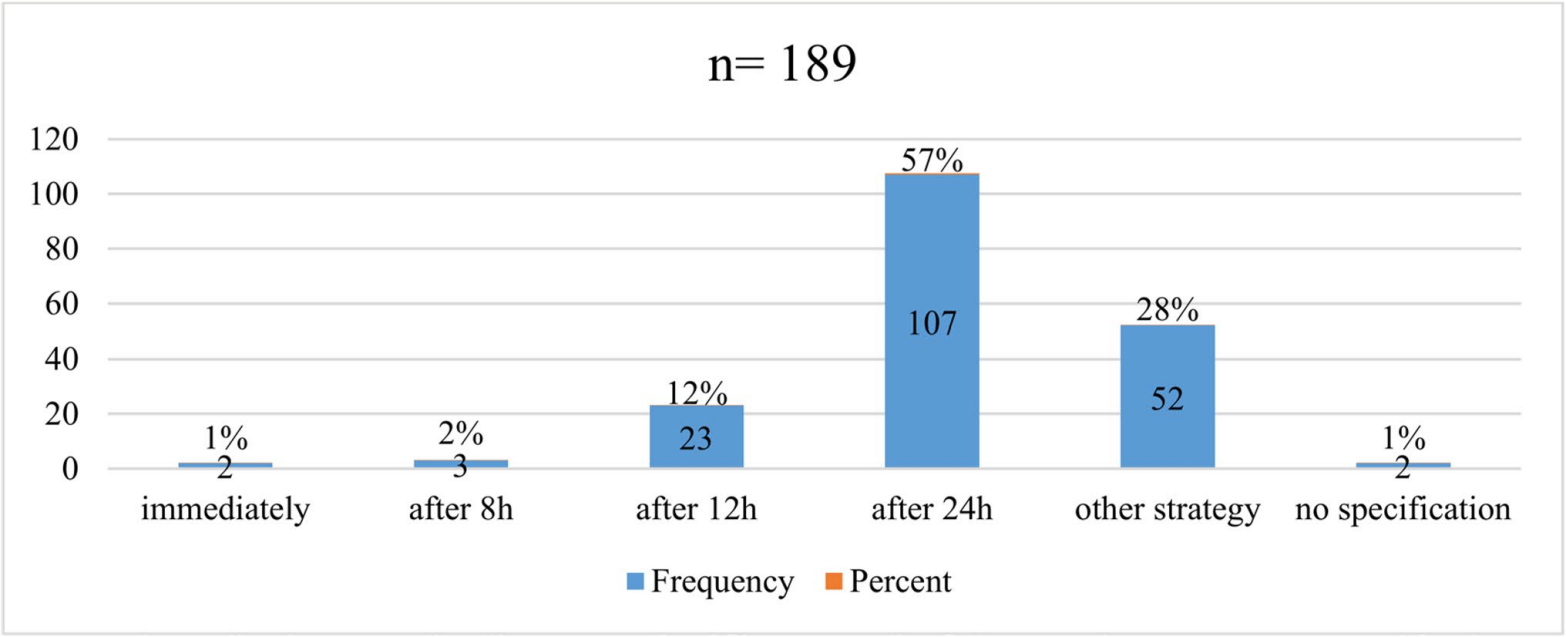
Time intervals after which the EP centers remove the pigtail catheter from the pericardium

Figure 6 shows the time intervals after which the EP centers remove the pigtail catheter from the pericardium. As the figure illustrates, 52/189 (28%) centers use a different strategy to remove the pigtail catheter. In 16/52 (31%) centers, the pigtail catheter is removed the following day, 36/52 (69%) responded to have other criteria for removing of the pigtail catheter, e.g., aspiration volume, echocardiographic control and catheter flow rate.

### Subsequent therapy

Two-thirds of the centers (128/189, 68%) stated that they do not routinely apply NSAIDs, colchicine or cortisone after pericardial tamponade. Medication applied by the other centers is provided in Suppl. Figure 9. A total of 87/189 (46%) centers cover their patients with antibiotics in case of pericardial tamponade and remaining pigtail catheter.

A total of 19/189 (10%) centers restart OAC after a certain number of hours, 48/189 (25%) after a certain time range after pericardial bleeding has stopped, and 84/189 (44%) after a certain number of hours following removal of the pigtail catheter. 35/189 (19%) centers chose a different strategy and 3/189 (2%) centers did not further specify (Suppl. Figure 10).

A total of 108/189 (57%) centers routinely schedule an outpatient follow-up visit in patients after cardiac tamponade, while 81/189 (43%) do not. 22/108 (20%) reported that they schedule their patients at day 7 after discharge. More than half (62/108, 57%) of the centers perform FU visits between day 7 and 14 and 13/108 (12%) centers later than 14 days post discharge. Another 4/108 (4%) centers report individualized approaches and 7/108 (6%) did not provide further information.

## Discussion

**Fig. 7:**
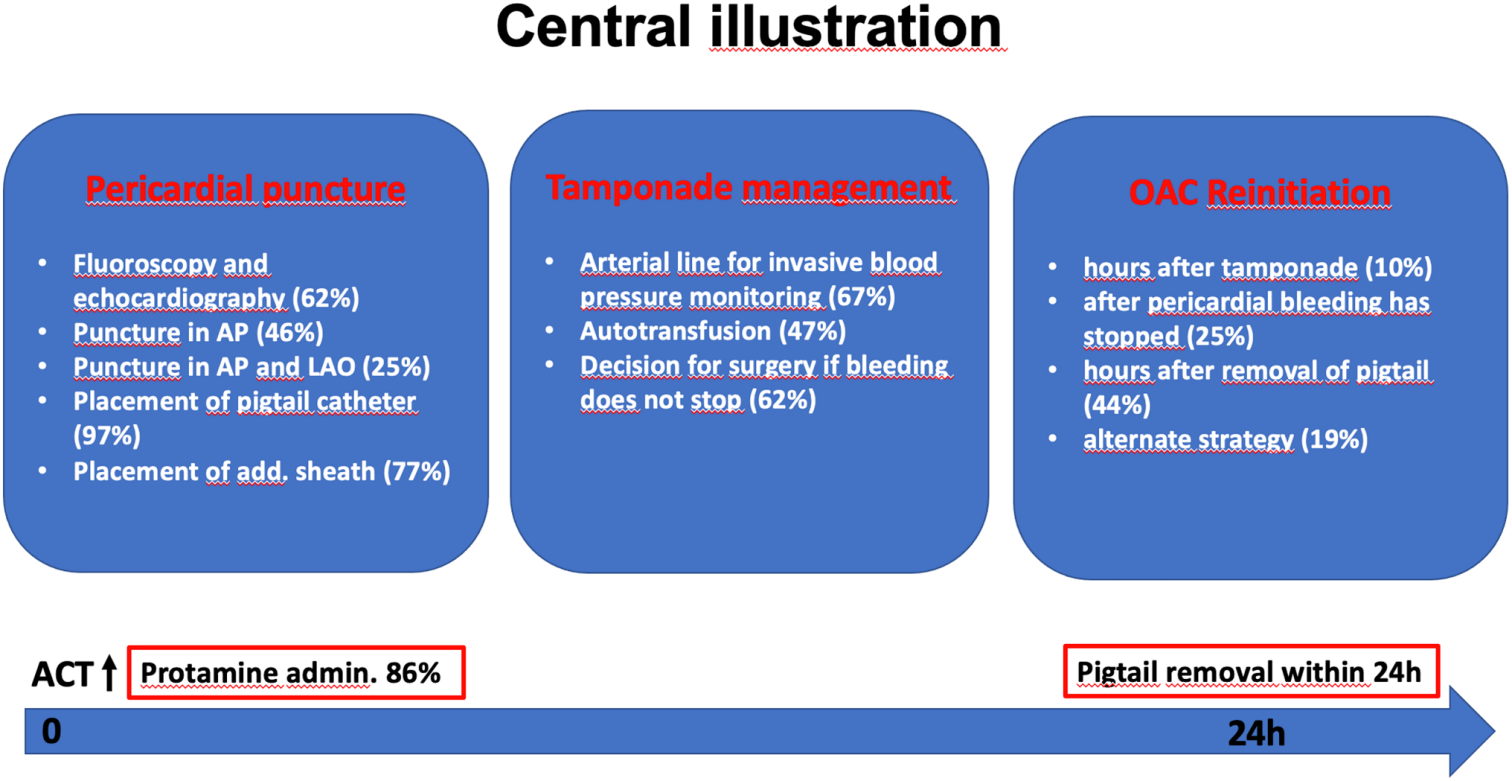
Central figure: typical aspects of management of cardiac tamponade. Shown is the administration of protamine to reverse the effect of heparin, pericardiocentesis and autotransfusion of aspirated blood, continued suction via a pigtail catheter for up to 24 h, and the gradual reinitiation of oral anticoagulation after tamponade

Tamponade is typically a consequence of an acute defect to the heart or intrapericardial vasculature, caused by chronic trauma (e.g., long-dwelling catheters in the right ventricular apex or in thin areas of the atria or the coronary sinus) or by acute damage, e.g., suboptimal transseptal puncture. These defects enabling bleeding into the pericardial sac typically close spontaneously, a process that can be delayed by anticoagulants. Robust evidence on the best management of tamponade is lacking, but most German EP centers have institutional standards for management of pericardial tamponade. Due to the lack of evidence, their workflows differ (Fig. 7). Most protocols include fluoroscopically guided pericardiocentesis, aspiration of pericardial blood via a pigtail catheter which is often inserted over a sheath, potentially autotransfusion of aspirated blood, invasive blood pressure monitoring, application of protamine in various forms and partly early involvement of cardiac surgery backup. A pigtail catheter is usually left in the pericardial space, typically for 24 h or less unless bleeding does not cease. After successful drainage, patients are monitored for at least 24 h. Approximately, half of the surveyed centers treat patients with antibiotics, NSAR and/or colchicine after tamponade.

The survey also identified several measures to reduce the risk of tamponade. Patient selection, mainly withholding ablation procedures in very old or very obese patients, is uncommon. Many centers have established routines for transseptal puncture, typically relying on fluoroscopic guidance. There is also a tendency to reduce the effect of anticoagulants by pausing of NOACs or by administering reduced doses of heparin prior to transseptal puncture.

### Patient selection and general precautions

Even though recommendations for the management of cardiac tamponade exist, major differences in patient handling before, during and after this emergency intervention are generally observed [5, 6].

One of the most important steps to prevent major complications during catheter ablation is patient selection and higher BMI, age and INR levels are considered as risk factors by physicians worldwide. In the present survey it is shown, that many German EP centers consider a BMI of 35 as a limiting factor for left atrial procedures or ablation of VTs with the majority of centers refusing to perform catheter ablation in patients with a BMI > 40. Importantly, although several reports have shown that catheter ablation can be safely performed in the elderly [8], [9–11], many centers in Germany discard catheter ablation in patients aged > 75 years and nearly all centers having participated in this survey refuse to do left atrial/ventricular procedures in octogenarians. Importantly, INR levels as a limiting factor were highly variable between the centers and some centers even accepted INR levels above 3.5 for left sided or VT ablation procedures. Thus, although some data exist regarding BMI, age and INR levels and their association to periinterventional risk, more robust data are obviously needed to enhance transparency.

### Puncture techniques of cardiac tamponade

Pericardial puncture in an emergency setting requires a high rate of flexibility and professionalism of the whole team and operators should rely on the technique and imaging modalities they are most trained on [12]. Most centers have a prepared emergency pericardial puncture set. The majority of centers prefer a pericardial puncture using both fluoroscopy and/or echocardiography, whereas a solely fluoroscopy-guided puncture or a solely echo-guided approach is less common. Most centers insert a pigtail catheter over a sheath and keep the pigtail catheter until the first postinterventional day. Larger dimensioned pigtail catheters (e.g., 7 F) might be advantageous as more volume can be aspirated in the same time from the pericardial space including smaller clots.

### Autotransfusion

The amount of aspirated blood or autotransfusion did not play a major role for clinical outcome in patients with cardiac tamponade in previous studies [5] but many centers in Germany obviously do autotransfusion. Autotransfusion is oftentimes performed without a filter, however, in some centers a filter set or even a cell saver is used. Autotransfusion might be of particular importance in massive bleeding from a left atrial or left ventricular defect or in general in cardiac tamponades requiring continuous aspiration for a longer time period. There is no general recommendation at which point-in-time autotransfusion should be considered in cardiac tamponade. However, autotransfusion can prevent the necessity of foreign blood transfusion and associated risks and can be performed immediately after pericardiocentesis.

### Anticoagulation management

Although uninterrupted NOAC therapy is recommended for patients undergoing AF ablation therapy, most centers participating in this survey withhold a least one dose at the day before or the day of ablation. However, this strategy also reflects lack on data of randomized controlled trials comparing truly versus minimally uninterrupted NOAC therapy in AF ablation [13].

Intraprocedural anticoagulation during cardiac tamponade might be crucial for a beneficial outcome. In the evaluated German centers, protamine is often administered on a routine basis in the setting of cardiac tamponade. However, the amount of protamine and the time point, when protamine is given, vary substantially. Furthermore, it has to be taken into consideration that protamine administration bears the risk of clot formation inside the pericardial space which might further complicate the acute situation or may increase the risk for late complications such as diastolic dysfunction. However, especially when not having cardiac surgery backup in house, protamine administration should be considered as early as possible.

Specific antidotes to NOACs have rarely been used in the surveyed centers to manage tamponade (in only 9/189 centers). Two-thirds of the centers would not apply specific NOAC antidotes, and one-fourth only in certain instances.

Despite robust data that left atrial ablation including transseptal puncture can be safely performed on continued NOAC therapy and without differences to uninterrupted vitamin K antagonist therapy [14–16], the majority of surveyed centers routinely pause NOAC therapy for 12–24 h prior to ablation procedures while continuing vitamin K antagonists [17]. This practice is reminiscent of other cardiovascular interventional teams, e.g., device or arterial interventions, that have traditionally paused oral anticoagulants. There is a clear need for a sufficiently powered randomized trial comparing interrupted and uninterrupted anticoagulation in patients undergoing ablation.

### Cardiac surgery

Cardiac surgery is usually considered as the last option to treat cardiac tamponade but is reported to be necessary in up to 12.8% of all cardiac tamponades occurring in the EP setting [5]. While cardiac tamponades during ablation of supraventricular tachycardias require cardiac surgery only in very rare instances, the necessity of surgical intervention in tamponades during left atrial procedures or ablation of VT is significantly higher [5, 6]. The optimal timing of cardiac surgery remains debatable as even in patients with large audible steam pops the location and severity of potential cardiac injury or perforation usually remains unclear. In patients with cardiac tamponade explored during cardiac surgery, a clear correlation to a specific cardiac injury is seen in only 14.2% in some populations, and in several patients, only diffuse bleeding is observed in situ [5]. The point in time when decision for open surgical exploration and treatment should be taken differs between the centers. If in-house surgical backup is provided, the decision for surgical intervention might be taken at a later stage since patient referral times are short.

### Postinterventional management

In hemodynamically stabilized patients after cardiac tamponade, the subsequent clinical management is of particular importance, as these patients are still at risk of death [5]. Thus, essential steps such as resumption of anticoagulation, withdrawal of the pigtail catheter and close echocardiographic reevaluation play a major role. The pigtail catheter is typically removed within 24 h after pericardiocentesis. In the majority of centers, anticoagulation is reinitiated after removal of the pigtail catheter. It should be considered, that stroke risk is significantly increased in particular after extensive LA ablation and smaller studies suggest, that early removement of the pericardial drain might be preferable [18]. Thus, reinitiation of OAC and removement of the pigtail catheter should be considered as early as possible after these procedures to prevent thromboembolic events.

### Limitations

The current analysis is based on a survey with its characteristic limitations. The survey was only conducted in Germany with response of roughly 50% of the addressed centers. The questionnaire was sent to all German cardiology centers offering EP-services. Since the questionnaire was anonymized, no information can be provided on which centers answered and which did not.

## Conclusion

The present survey shows that the management of cardiac tamponade is still inhomogeneous in German ablation centers. However, multiple results and aspects of that survey can be generalized and might guide especially less experienced operators and centers in their treatment and decision strategies when acute pericardial tamponade occurs. It might be of value to implement a position paper focusing on the management of cardiac tamponade.

## Data Availability

Data will be available on reasonable request.
