# Correction to: Management of pericardial tamponade in the electrophysiology laboratory: results from a national survey

**DOI:** 10.1007/s00392-022-02066-3

**Published:** 2022-08-04

**Authors:** Andreas Metzner, Stephan D. Reubold, Sophie Schönhofer, Bruno Reißmann, Feifan Ouyang, Laura Rottner, Ruben Schleberger, Leon Dinshaw, Julia Moser, Fabian Moser, Marc Lemoine, Paula Münkler, Shinwan Kany, Daniel Steven, Philipp Sommer, Paulus Kirchhof, Andreas Rillig

**Affiliations:** 1 Department of Cardiology, University Heart and Vascular Center Hamburg, Martinistraße 52, 20246 Hamburg, Germany; 2 DZHK, Hamburg, Germany; 3 DZHK, Kiel, Germany; 4 DZHK, Lübeck, Germany; 5 University Heart Center, University of Cologne, Cologne, Germany; 6 Herz- Und Diabeteszentrum NRW, Bad Oeynhausen, Ruhr-University of Bochum, Bad Oeynhausen, Germany; 7 Institute of Cardiovascular Sciences, University of Birmingham, Birmingham, UK; 8 Asklepios Klinik Harburg, Hamburg, Germany

## Correction to: Clinical Research in Cardiology 10.1007/s00392-022-02042-x

In this article, the author’s name Shinwan Kany was incorrectly given as Shino Kany.

The original article has been corrected.

